# Supplementary material for: Prediction of Small for Gestational Age and Growth-Restricted Neonates at 35 to 36 Weeks of Gestation: A Multicenter Cohort Study
Source: Medicina (Kaunas). 2025 Sep 8;61(9):1626. doi: 10.3390/medicina61091626 (PMC12471666; doi:10.3390/medicina61091626)

Supplemental Figure S1. Calibration plots for prediction of birthweight below the 10th centile. The black diagonal line representes perfect agreement. Circles and vertical lines represent the observed incidence with 95% CI. Numbers at the left are cases of birthweight below the 10th centile and numbers at the right are the number of cases in each predicted risk category.

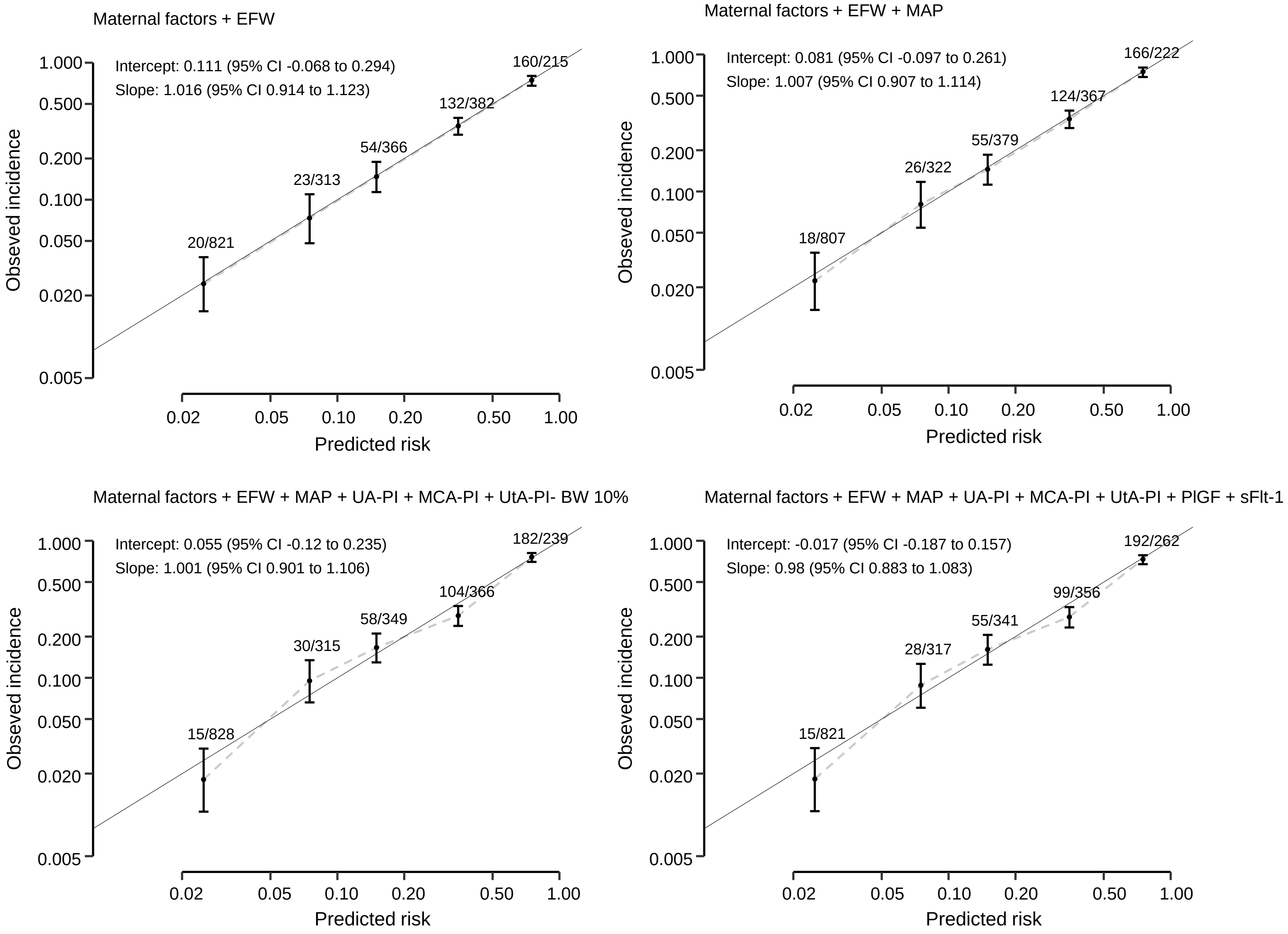

Supplemental Figure S2. Calibration plots for prediction of birthweight below the 5th centile. The black diagonal line representes perfect agreement. Circles and vertical lines represent the observed incidence with 95% CI. Numbers at the left are cases of birthweight below the 5th centile and numbers at the right are the number of cases in each predicted risk category.

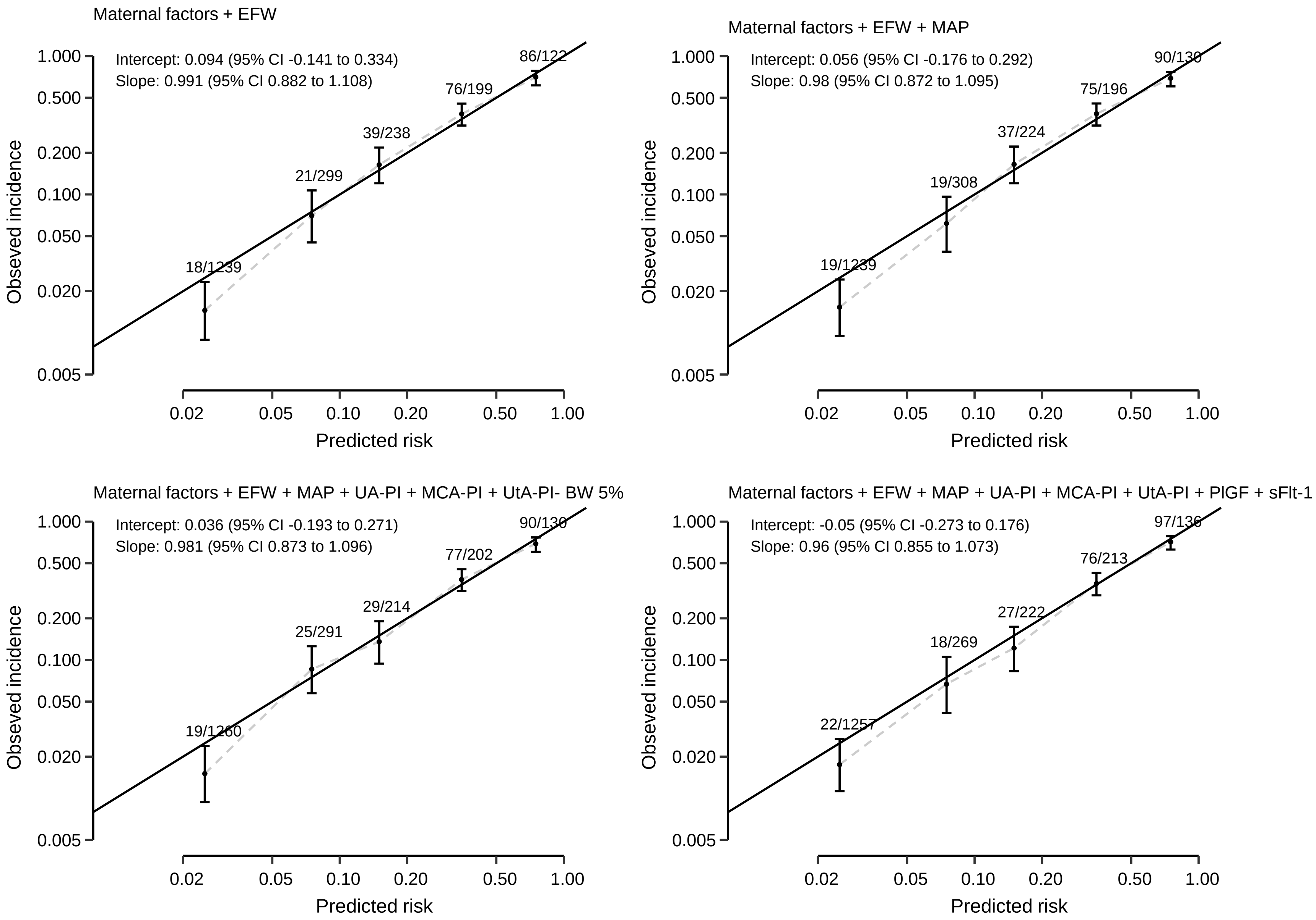

Supplement: Supplementary file 1 [file medicina-61-01626-s001.zip › medicina-3842214-supplementary/supplementary/medicina-3842214-figures.pdf]
